# 2,2′-Dithio­dianiline: a redetermination at 100 K

**DOI:** 10.1107/S1600536810000024

**Published:** 2010-01-09

**Authors:** Jia Hao Goh, Hoong-Kun Fun, M. Babu, B. Kalluraya

**Affiliations:** a X-ray Crystallography Unit, School of Physics, Universiti Sains Malaysia, 11800 USM, Penang, Malaysia; b Department of Studies in Chemistry, Mangalore University, Mangalagangotri, Mangalore 574 199, India

## Abstract

Structural studies of the title compound [systematic name: 2,2′-(disulfanedi­yl)dianiline], C_12_H_12_N_2_S_2_, were previously performed at room temperature [Gomes de Mesquita (1967 ▶). *Acta Cryst.* **23**, 671; Lee & Bryant (1970 ▶). *Acta Cryst.* B**26**, 1729; Ribar *et al.* (1975 ▶). *Bull. Yugoslav. Crystallogr. Centre*, A**10**, 68]. The results of the current redetermination allow a clarification of the nature of the intra- and inter­molecular N—H⋯S hydrogen bonding described in the literature for this compound. On cooling to 100 K, the unit cell contracts most in the *c* axis, and it changes rather less in the directions involving the strongly hydrogen-bonded chains, which are the *a* and *b* axes. In the crystal structure, N—H⋯N hydrogen bonds link neighbouring mol­ecules into two-dimensional frameworks parallel to the *ab* plane. An additional inter­molecular N—H⋯S hydrogen bond has also been established, based on freely refined H-atom positions. Inter­molecular C—H⋯π inter­actions further stabilize the crystal structure.

## Related literature

For previously reported structure determinations of the title compound, see: Gomes de Mesquita (1967 ▶); Lee & Bryant (1970 ▶); Ribar *et al.* (1975 ▶). For general background to and applications of the title compound, see: Garbarczyk *et al.* (1999 ▶); Kalluraya *et al.* (2000 ▶); Kalluraya & Chimbalkar (2001 ▶). For a description of the Cambridge Structural Database, see: Allen (2002 ▶). For the stability of the temperature controller used for the data collection, see: Cosier & Glazer (1986 ▶).
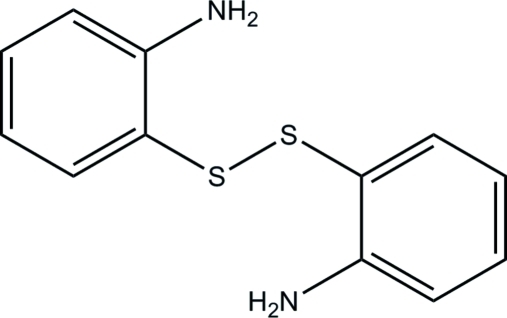


## Experimental

### 

#### Crystal data

C_12_H_12_N_2_S_2_
*M* *_r_* = 248.36
Orthorhombic, 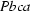

*a* = 8.2531 (1) Å
*b* = 13.0278 (2) Å
*c* = 22.3655 (4) Å
*V* = 2404.73 (6) Å^3^
*Z* = 8
Mo *K*α radiation
μ = 0.42 mm^−1^
*T* = 100 K
0.23 × 0.18 × 0.17 mm

#### Data collection

Bruker SMART APEXII CCD area-detector diffractometer
Absorption correction: multi-scan (*SADABS*; Bruker, 2009 ▶) *T*_min_ = 0.912, *T*_max_ = 0.934
47724 measured reflections
2750 independent reflections
2417 reflections with *I* > 2σ(*I*)
*R*_int_ = 0.040

#### Refinement

*R*[*F* ^2^ > 2σ(*F* ^2^)] = 0.048
*wR*(*F* ^2^) = 0.124
*S* = 1.19
2750 reflections
193 parameters
All H-atom parameters refined
Δρ_max_ = 0.50 e Å^−3^
Δρ_min_ = −0.43 e Å^−3^

### 

Data collection: *APEX2* (Bruker, 2009 ▶); cell refinement: *SAINT* (Bruker, 2009 ▶); data reduction: *SAINT*; program(s) used to solve structure: *SHELXTL* (Sheldrick, 2008 ▶); program(s) used to refine structure: *SHELXTL*; molecular graphics: *SHELXTL*; software used to prepare material for publication: *SHELXTL* and *PLATON* (Spek, 2009 ▶).

## Supplementary Material

Crystal structure: contains datablocks global, I. DOI: 10.1107/S1600536810000024/tk2598sup1.cif

Structure factors: contains datablocks I. DOI: 10.1107/S1600536810000024/tk2598Isup2.hkl

Additional supplementary materials:  crystallographic information; 3D view; checkCIF report

## Figures and Tables

**Table 1:** Hydrogen-bond geometry (Å, °) *Cg*1 and *Cg*2 are the centroids of the C7–C12 and C1–C16 phenyl rings, respectively.

*D*—H⋯*A*	*D*—H	H⋯*A*	*D*⋯*A*	*D*—H⋯*A*
N1—H1*N*1⋯N2^i^	0.87 (4)	2.39 (4)	3.222 (3)	160 (4)
N2—H1*N*2⋯N1^ii^	0.86 (4)	2.39 (4)	3.184 (3)	155 (3)
N2—H2*N*2⋯S1^iii^	0.88 (4)	2.61 (3)	3.436 (2)	156 (3)
C4—H4*A*⋯*Cg*1^iv^	0.97 (3)	2.95 (3)	3.689 (3)	134 (2)
C9—H9*A*⋯*Cg*2^v^	0.96 (3)	2.89 (3)	3.637 (3)	135 (2)

Symmetry codes: (i) 
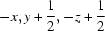
; (ii) 
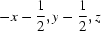
; (iii) 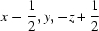; (iv) 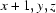; (v) 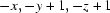.

**Fig. 1.**
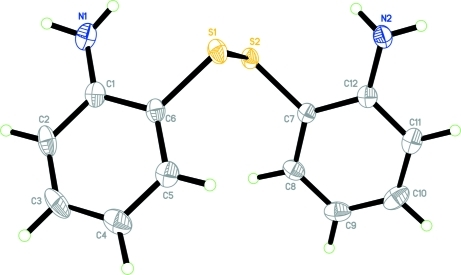
The molecular structure of the title compound, showing 50% probability displacement ellipsoids for non-H atoms and the atom-numbering scheme.

**Fig. 2.**
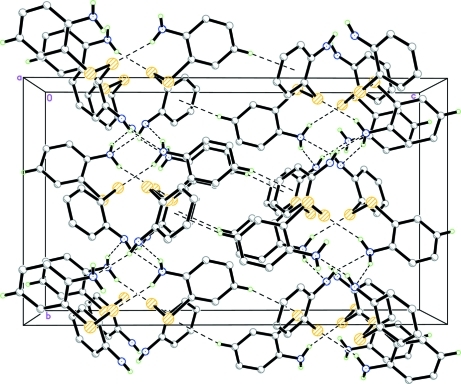
Part of the crystal structure, viewed along the a axis, showing two interlayers being joined along the c axis by weak intermolecular C—H···π interactions. Hydrogen atoms not involved in intermolecular interactions (dashed lines) have been omitted for clarity.

**Fig. 3.**
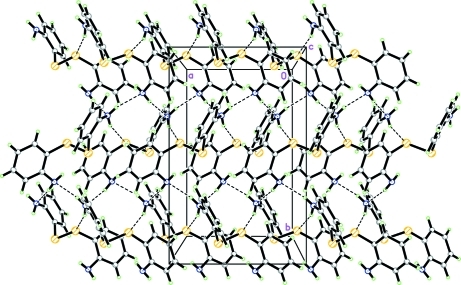
The crystal structure of the title compound, viewed along the c axis, showing a two-dimensional framework parallel to the ab plane. Intermolecular hydrogen bonds are shown as dashed lines.

## supplementary crystallographic information

### Comment

Nitrogen- and sulphur- containing compounds are important intermediates in the synthesis of various heterocyclic compounds. Ortho amino thiophenols are important precursors in the preparation of a variety of heterocycles such as benzothiadiazepines, thiadiazoles, thiadiazines *etc.* (Kalluraya *et al.*, 2000; Kalluraya & Chimbalkar, 2001). The title compound was obtained during an attempt to study the aerial oxidation of aminothiophenols. Molecular and crystal structures of thioamide derivatives have been analyzed (Garbarczyk *et al.*, 1999).

A search of the November 2008 release of the Cambridge Structural Database (Allen, 2002) reveals that the room temperature crystal structures of the title compound (Fig. 1) were first reported with *R* = 0.057 for 369 reflections (Gomes de Mesquita, 1967), then followed with *R* = 0.086 for 1313 reflections (Lee & Bryant, 1970) and *R* = 0.042 (Ribar *et al.*, 1975). The current redetermination at 100 K increases significantly the precision of the structural and geometrical parameters and provides a lower *R* value (*R* = 0.048 based on 2750 independent observed reflections).

Comparison with the previously reported unit cell parameters (Ribar *et al.*, 1975) reveals that on cooling to 100 K, *a* expands by 0.41 %, whereas *b* and *c* contract by 1.15 and 1.74 %, respectively. This can be explained by the fact that along the *c *axis, the molecules are interconnected by weak C—H···π interactions only whereas along the *a* and *b* axes, the molecules are interconnected by the stronger hydrogen bonds (Figs 2 & 3, Table 1). The previously reported structure (Lee & Bryant, 1970) suggests two intramolecular N—H···S hydrogen bonds, but the current work observes no such intramolecular hydrogen bonds with bond angle larger than 120°. The bond lengths are comparable to but more precise than the previously reported structures (Lee & Bryant, 1970; Ribar *et al.*, 1975).

In the crystal structure, molecules are linked into two-dimensional frameworks parallel to the *ab* plane (Fig. 3) rather than one-dimensional infinite chains as reported previously (Lee & Bryant, 1970). Based on freely refined hydrogen atom positions, an additional intermolecular N2—H2N2···S1 hydrogen bond (Table 1) has also been established. Intermolecular C4—H4A···*Cg*1 and C9—H9A···*Cg*2 interactions (Table 1) further stabilize the crystal structure.

### Experimental

The title compound is obtained by exposing 2-aminobenzenethiol to sunlight in an open beaker for two days. The reagent 2-aminobenzenethiol undergoes self aerial oxidation to furnish the crystals. The crude product obtained through the photochemical condition was washed with ethanol and dried. Single crystals suitable for X-ray analysis were obtained from ethanol by slow evaporation.

### Refinement

All the H atoms were located from difference Fourier map [range of C—H = 0.93 (3) - 1.00 (3) Å, and see Table 1 for N–H distances] and allowed to refine freely.

### Crystal data

**Table:**

C_12_H_12_N_2_S_2_	*F*(000) = 1040
*M**_r_* = 248.36	*D*_x_ = 1.372 Mg m^−^^3^
Orthorhombic, *P**b**c**a*	Mo *K*α radiation, λ = 0.71073 Å
Hall symbol: -P 2ac 2ab	Cell parameters from 9179 reflections
*a* = 8.2531 (1) Å	θ = 3.1–29.9°
*b* = 13.0278 (2) Å	µ = 0.42 mm^−^^1^
*c* = 22.3655 (4) Å	*T* = 100 K
*V* = 2404.73 (6) Å^3^	Block, yellow
*Z* = 8	0.23 × 0.18 × 0.17 mm

### Data collection

**Table:**

Bruker SMART APEXII CCD area-detector diffractometer	2750 independent reflections
Radiation source: fine-focus sealed tube	2417 reflections with *I* > 2σ(*I*)
graphite	*R*_int_ = 0.040
φ and ω scans	θ_max_ = 27.5°, θ_min_ = 3.1°
Absorption correction: multi-scan (*SADABS*; Bruker, 2009)	*h* = −10→10
*T*_min_ = 0.912, *T*_max_ = 0.934	*k* = −16→16
47724 measured reflections	*l* = −29→29

### Refinement

**Table:**

Refinement on *F*^2^	Primary atom site location: structure-invariant direct methods
Least-squares matrix: full	Secondary atom site location: difference Fourier map
*R*[*F*^2^ > 2σ(*F*^2^)] = 0.048	Hydrogen site location: inferred from neighbouring sites
*wR*(*F*^2^) = 0.124	All H-atom parameters refined
*S* = 1.19	*w* = 1/[σ^2^(*F*_o_^2^) + (0.0383*P*)^2^ + 5.2113*P*] where *P* = (*F*_o_^2^ + 2*F*_c_^2^)/3
2750 reflections	(Δ/σ)_max_ < 0.001
193 parameters	Δρ_max_ = 0.50 e Å^−^^3^
0 restraints	Δρ_min_ = −0.43 e Å^−^^3^

### Special details

**Table:**

Experimental. The crystal was placed in the cold stream of an Oxford Cyrosystems Cobra open-flow nitrogen cryostat (Cosier & Glazer, 1986) operating at 100.0 (1)K.
Geometry. All esds (except the esd in the dihedral angle between two l.s. planes) are estimated using the full covariance matrix. The cell esds are taken into account individually in the estimation of esds in distances, angles and torsion angles; correlations between esds in cell parameters are only used when they are defined by crystal symmetry. An approximate (isotropic) treatment of cell esds is used for estimating esds involving l.s. planes.
Refinement. Refinement of F^2^ against ALL reflections. The weighted R-factor wR and goodness of fit S are based on F^2^, conventional R-factors R are based on F, with F set to zero for negative F^2^. The threshold expression of F^2^ > 2sigma(F^2^) is used only for calculating R-factors(gt) etc. and is not relevant to the choice of reflections for refinement. R-factors based on F^2^ are statistically about twice as large as those based on F, and R- factors based on ALL data will be even larger.

### Fractional atomic coordinates and isotropic or equivalent isotropic displacement parameters (Å^2^)

**Table:**

	*x*	*y*	*z*	*U*_iso_*/*U*_eq_	
S1	0.02244 (8)	0.44246 (5)	0.29158 (3)	0.02175 (17)	
S2	−0.18075 (7)	0.49532 (5)	0.33614 (3)	0.01897 (17)	
N1	0.1434 (3)	0.65943 (19)	0.27327 (11)	0.0261 (5)	
N2	−0.3466 (3)	0.28926 (19)	0.32376 (10)	0.0226 (5)	
C1	0.2321 (3)	0.6022 (2)	0.31445 (12)	0.0208 (5)	
C2	0.3664 (3)	0.6450 (2)	0.34391 (13)	0.0267 (6)	
C3	0.4491 (3)	0.5893 (2)	0.38682 (14)	0.0308 (7)	
C4	0.4032 (3)	0.4902 (2)	0.40210 (13)	0.0287 (6)	
C5	0.2712 (3)	0.4467 (2)	0.37320 (12)	0.0233 (5)	
C6	0.1857 (3)	0.5017 (2)	0.32969 (11)	0.0193 (5)	
C7	−0.2077 (3)	0.40351 (19)	0.39331 (11)	0.0171 (5)	
C8	−0.1543 (3)	0.4247 (2)	0.45123 (12)	0.0220 (5)	
C9	−0.1837 (3)	0.3567 (2)	0.49755 (12)	0.0275 (6)	
C10	−0.2661 (4)	0.2664 (2)	0.48554 (13)	0.0295 (6)	
C11	−0.3206 (3)	0.2437 (2)	0.42842 (13)	0.0251 (6)	
C12	−0.2934 (3)	0.31171 (19)	0.38114 (11)	0.0190 (5)	
H2A	0.402 (4)	0.715 (3)	0.3335 (14)	0.034 (9)*	
H3A	0.536 (4)	0.621 (3)	0.4078 (15)	0.038 (9)*	
H4A	0.457 (4)	0.451 (3)	0.4337 (15)	0.033 (9)*	
H5A	0.240 (4)	0.379 (2)	0.3819 (13)	0.025 (8)*	
H8A	−0.100 (4)	0.492 (2)	0.4590 (14)	0.027 (8)*	
H9A	−0.153 (4)	0.373 (2)	0.5377 (14)	0.022 (8)*	
H10A	−0.285 (4)	0.219 (3)	0.5158 (14)	0.030 (9)*	
H11A	−0.374 (4)	0.179 (3)	0.4193 (14)	0.029 (8)*	
H1N1	0.199 (5)	0.706 (3)	0.2542 (18)	0.048 (11)*	
H2N1	0.083 (5)	0.620 (3)	0.2483 (16)	0.040 (10)*	
H1N2	−0.422 (5)	0.244 (3)	0.3209 (15)	0.037 (9)*	
H2N2	−0.362 (4)	0.343 (3)	0.3004 (14)	0.025 (8)*	

### Atomic displacement parameters (Å^2^)

**Table:**

	*U*^11^	*U*^22^	*U*^33^	*U*^12^	*U*^13^	*U*^23^
S1	0.0167 (3)	0.0245 (3)	0.0240 (3)	−0.0045 (2)	0.0039 (2)	−0.0044 (3)
S2	0.0123 (3)	0.0188 (3)	0.0258 (3)	−0.0004 (2)	0.0000 (2)	0.0029 (2)
N1	0.0241 (12)	0.0220 (12)	0.0321 (13)	0.0011 (10)	0.0068 (10)	0.0039 (10)
N2	0.0204 (11)	0.0205 (11)	0.0268 (12)	−0.0038 (9)	−0.0012 (9)	−0.0006 (9)
C1	0.0160 (12)	0.0212 (12)	0.0253 (12)	0.0025 (10)	0.0079 (10)	−0.0026 (10)
C2	0.0163 (12)	0.0258 (14)	0.0381 (15)	−0.0049 (11)	0.0101 (11)	−0.0089 (12)
C3	0.0112 (12)	0.0430 (17)	0.0382 (16)	−0.0015 (12)	0.0018 (11)	−0.0154 (13)
C4	0.0152 (12)	0.0395 (17)	0.0313 (15)	0.0069 (11)	0.0002 (11)	−0.0045 (13)
C5	0.0185 (12)	0.0235 (13)	0.0278 (13)	0.0035 (10)	0.0050 (10)	−0.0007 (11)
C6	0.0140 (11)	0.0219 (12)	0.0220 (12)	−0.0008 (9)	0.0057 (9)	−0.0013 (10)
C7	0.0100 (11)	0.0188 (12)	0.0225 (12)	0.0029 (9)	0.0026 (9)	0.0022 (9)
C8	0.0144 (12)	0.0275 (14)	0.0242 (13)	0.0028 (10)	−0.0004 (10)	−0.0030 (10)
C9	0.0211 (13)	0.0400 (17)	0.0214 (13)	0.0066 (12)	0.0004 (10)	0.0007 (12)
C10	0.0278 (14)	0.0325 (16)	0.0282 (14)	0.0090 (12)	0.0086 (12)	0.0095 (12)
C11	0.0214 (13)	0.0206 (13)	0.0333 (14)	0.0022 (11)	0.0068 (11)	0.0029 (11)
C12	0.0114 (11)	0.0212 (12)	0.0244 (12)	0.0028 (9)	0.0038 (9)	−0.0009 (10)

### Geometric parameters (Å, °)

**Table:**

S1—C6	1.771 (3)	C4—C5	1.388 (4)
S1—S2	2.0687 (9)	C4—H4A	0.97 (3)
S2—C7	1.765 (3)	C5—C6	1.400 (4)
N1—C1	1.392 (4)	C5—H5A	0.94 (3)
N1—H1N1	0.87 (4)	C7—C8	1.396 (4)
N1—H2N1	0.91 (4)	C7—C12	1.416 (3)
N2—C12	1.388 (3)	C8—C9	1.384 (4)
N2—H1N2	0.86 (4)	C8—H8A	1.00 (3)
N2—H2N2	0.88 (3)	C9—C10	1.386 (4)
C1—C2	1.405 (4)	C9—H9A	0.96 (3)
C1—C6	1.407 (4)	C10—C11	1.386 (4)
C2—C3	1.383 (4)	C10—H10A	0.93 (3)
C2—H2A	0.98 (3)	C11—C12	1.397 (4)
C3—C4	1.388 (4)	C11—H11A	0.97 (3)
C3—H3A	0.95 (4)		
			
C6—S1—S2	103.87 (9)	C6—C5—H5A	119 (2)
C7—S2—S1	103.05 (8)	C5—C6—C1	120.5 (2)
C1—N1—H1N1	115 (3)	C5—C6—S1	119.7 (2)
C1—N1—H2N1	113 (2)	C1—C6—S1	119.8 (2)
H1N1—N1—H2N1	112 (3)	C8—C7—C12	120.2 (2)
C12—N2—H1N2	116 (2)	C8—C7—S2	119.9 (2)
C12—N2—H2N2	115 (2)	C12—C7—S2	119.74 (19)
H1N2—N2—H2N2	113 (3)	C9—C8—C7	120.9 (3)
N1—C1—C2	120.8 (3)	C9—C8—H8A	120.6 (18)
N1—C1—C6	121.0 (2)	C7—C8—H8A	118.4 (18)
C2—C1—C6	118.1 (3)	C8—C9—C10	119.0 (3)
C3—C2—C1	120.4 (3)	C8—C9—H9A	121.3 (18)
C3—C2—H2A	120 (2)	C10—C9—H9A	119.7 (18)
C1—C2—H2A	119 (2)	C9—C10—C11	121.2 (3)
C2—C3—C4	121.6 (3)	C9—C10—H10A	121 (2)
C2—C3—H3A	119 (2)	C11—C10—H10A	118 (2)
C4—C3—H3A	119 (2)	C10—C11—C12	120.7 (3)
C5—C4—C3	118.7 (3)	C10—C11—H11A	121.7 (19)
C5—C4—H4A	119 (2)	C12—C11—H11A	117.6 (19)
C3—C4—H4A	122 (2)	N2—C12—C11	121.0 (2)
C4—C5—C6	120.7 (3)	N2—C12—C7	120.9 (2)
C4—C5—H5A	120 (2)	C11—C12—C7	118.0 (2)
			
C6—S1—S2—C7	−89.29 (13)	S1—S2—C7—C8	99.8 (2)
N1—C1—C2—C3	176.9 (2)	S1—S2—C7—C12	−84.50 (19)
C6—C1—C2—C3	−0.4 (4)	C12—C7—C8—C9	0.1 (4)
C1—C2—C3—C4	0.1 (4)	S2—C7—C8—C9	175.7 (2)
C2—C3—C4—C5	0.2 (4)	C7—C8—C9—C10	0.6 (4)
C3—C4—C5—C6	−0.3 (4)	C8—C9—C10—C11	−0.7 (4)
C4—C5—C6—C1	0.0 (4)	C9—C10—C11—C12	0.1 (4)
C4—C5—C6—S1	177.7 (2)	C10—C11—C12—N2	179.6 (3)
N1—C1—C6—C5	−177.0 (2)	C10—C11—C12—C7	0.5 (4)
C2—C1—C6—C5	0.3 (4)	C8—C7—C12—N2	−179.7 (2)
N1—C1—C6—S1	5.4 (3)	S2—C7—C12—N2	4.7 (3)
C2—C1—C6—S1	−177.36 (19)	C8—C7—C12—C11	−0.6 (3)
S2—S1—C6—C5	98.6 (2)	S2—C7—C12—C11	−176.27 (19)
S2—S1—C6—C1	−83.8 (2)		

### Hydrogen-bond geometry (Å, °)

**Table:**

Cg1 and Cg2 are the centroids for C7–C12 and C1–C16 phenyl rings, respectively.

**Table:**

*D*—H···*A*	*D*—H	H···*A*	*D*···*A*	*D*—H···*A*
N1—H1N1···N2^i^	0.87 (4)	2.39 (4)	3.222 (3)	160 (4)
N2—H1N2···N1^ii^	0.86 (4)	2.39 (4)	3.184 (3)	155 (3)
N2—H2N2···S1^iii^	0.88 (4)	2.61 (3)	3.436 (2)	156 (3)
C4—H4A···Cg1^iv^	0.97 (3)	2.95 (3)	3.689 (3)	134 (2)
C9—H9A···Cg2^v^	0.96 (3)	2.89 (3)	3.637 (3)	135 (2)

Symmetry codes: (i) −*x*, *y*+1/2, −*z*+1/2; (ii) −*x*−1/2, *y*−1/2, *z*; (iii) *x*−1/2, *y*, −*z*+1/2; (iv) *x*+1, *y*, *z*; (v) −*x*, −*y*+1, −*z*+1.
